# Dataset for corruption risk assessment in a public administration

**DOI:** 10.1016/j.dib.2021.107768

**Published:** 2021-12-29

**Authors:** Marcelo Oliveira Vasconcelos, Luís Cavique

**Affiliations:** a Tribunal de Contas do Distrito Federal, Brasília, Brazil; b Universidade Aberta, Lisboa, Portugal; c LASIGE, Lisboa, Portugal

**Keywords:** Data enrichment, Imbalanced learning, Corruption, Public administration, Risk

## Abstract

This data article describes a dataset of corruption approach and possible variables related, and this dataset was created by integrating eight different systems of Brazilian federal government and Federal District. We present real data from civil servants and militaries to comply with GDPR legislation, the attributes that could identify a person were removed, making the data anonymized.

**Specifications Table**

**Table:** 

Subject	Information Systems and Management
Specific subject area	Corruption, civil servant, logistic regression
Type of data	Table (csv file)
How data were acquired	Data were acquired from eight different databases from The Brazilian Government with *SAS Enterprise Guide*
Data format	Mixed (raw and pre-processed)
Parameters for data collection	This dataset corresponds to data available in November 2020 and refers to all civil servants and militaries in Federal District.
Description of data collection	A compilation of Brazilian government databases was used for this research and integrated eight databases from Federal Government and Federal District related by sanctions, Civil Service Payment Systems, Political and Firms/Companies.
Data source location	Federal District, Brazil https://dados.gov.br/dataset?_organization_limit=0 Institutions: Controladoria-Geral da União – CGU, https://dadosabertos.tse.jus.br/ http://www.transparencia.df.gov.br/#/https://www.gov.br/receitafederal/pt-br/acesso-a-informacao/dados-abertoshttps://www2.tc.df.gov.br/controle-externo/inabilitados-para-cargos-em-comissao/
Data accessibility	Repository name: Dataset for corruption risk assessment in a Public Administration Data identification number: doi:10.17632/crpdknzswh.2 Direct link to the dataset: https://data.mendeley.com/datasets/crpdknzswh/2
Related research article	Vasconcelos, M. O., Chaim, R. M., & Cavique, L. (2021). Imbalanced Learning in Assessing the Risk of Corruption in Public Administration BT - Progress in Artificial Intelligence (G. Marreiros, F. S. Melo, N. Lau, H. Lopes Cardoso, & L. P. Reis (eds.); pp. 510–523). Springer International Publishing.

**Value of the Data**

• This dataset contains data from eight different databases from the Brazilian federal government and Federal District.
• This dataset benefits researchers working in the field of corruption risk assessment and also applied machine learning.
• Researchers working in the field of corruption risk assessment may find this dataset benefited and could also apply machine learning.
• The analysis of this data could help identify corruption risk factors and assist in the definition of overseen planning on focus on the activities of the greatest risk for Public Administration, such as cases with a high probability of occurrence and a high financial or social impact.

## 1 Data Description

The dataset provided in this paper offers valuable information on public administration and allows research in the corruption area. A few datasets regarding corruption are available, Al-Jundi [1] presents a survey dataset on determinants of administrative corruption, Peerthum et al. [2] related to corruption in Mauritius, and Oguntunde et al. [3] deal with selected crime data in Nigeria, including corruption.

Literature was consulted to determine attributes for administrative corruption. Other researchers can reuse the dataset and can be easily downloaded from the Mendeley Data repository.^1^

The data in this article are composed of all civil servants from Federal District Government (Brazilian Public Administration), involves the reported cases of dismission by corruption, and aggregate 26 attributes related to four domain areas extract from eight databases.

These four domains are related by sources provided and are:

• Corruption Domain (C) aggregate data corresponding to illegal acts committed by civil servants or militaries or companies that they are owners;
• Employment domain (E) provide servant's registrations from Human Resources Management System like income and number of coordination roles;
• Political Domain (P) covers data related to political activities; and
• Business Domain (B) presents company features that civil servants and militaries are owners.

### 1.1 The descriptive statistics

The dataset is composed of 27 attributes, part of them are integer and numeric attributes (Table 2), other attributes are categorical (Table 3), and a few of them are Boolean (Table 4).

All boolean attributes (Table 4) belong to Corruption Domain.

Table 3 presents categorical attributes from Political and Business Domains, and Table 2 shows the main statistic description from Employment and Business domains with integer or numeric attributes.

## 2 Experimental Design, Materials and Methods

This section gives Data Sources aggregated information; Related Literature to compose the dataset features, Descriptive Statistics, and the Preprocessing (Data Enrichment and Data Cleansing).

### 2.1 Data sources

The dataset was composed of eight different sources from Brazilian public administration. After consolidation, the attributes were classified by four domain areas for better understand, described by: corruption(C), Employment (E), political (P), and Business (B) that are related by sources.

The dataset was created after an ETL process collected from these different data sources:

• CGU-CEPIM - Private Non-Profit Entities Prevented from contracting with the Public Administration maintained by Office of the Comptroller General (Controladoria-Geral da União—CGU);
• CGU-CEIS - Registration of Unfaithful and Suspended Companies) maintained by Office of the Comptroller General (Controladoria-Geral da União—CGU);
• CGDF - Expulsion Registrations maintained by Comptroller General of the Federal District (Portal da Transparência DF);
• TCDF - Persons that by sanction are not allowed for the exercise commission position or a trust function within the scope of the Public Administration of the Federal District maintained by District Federal Court of Accounts– TCDF;
• SIAPE – Integrated Human Resources Administration System maintained by Federal Government;
• SIGRH - Integrated Resource Management System maintained by Federal District Government;
• TSE- Electoral Data maintained by Superior Electoral Court (TSE); and
• SRF/ME - Personal and Legal Data maintained by Secretariat of Brazil's Federal Revenue (SRF/ME).

These data represent the information from civil servants, militaries, and pensioners of The Federal District, a Brazilian Public Administration, in total are 303,036.

Federal District is a legal entity of internal public law, which is part of the political-administrative structure of Brazil, of a nature sui generis, because it is neither a state nor a municipality, but a special entity that accumulates the legislative powers reserved to the states and the municipalities, which gives it a hybrid nature of state and municipality.

### 2.2 Domains

**Fig 1:**
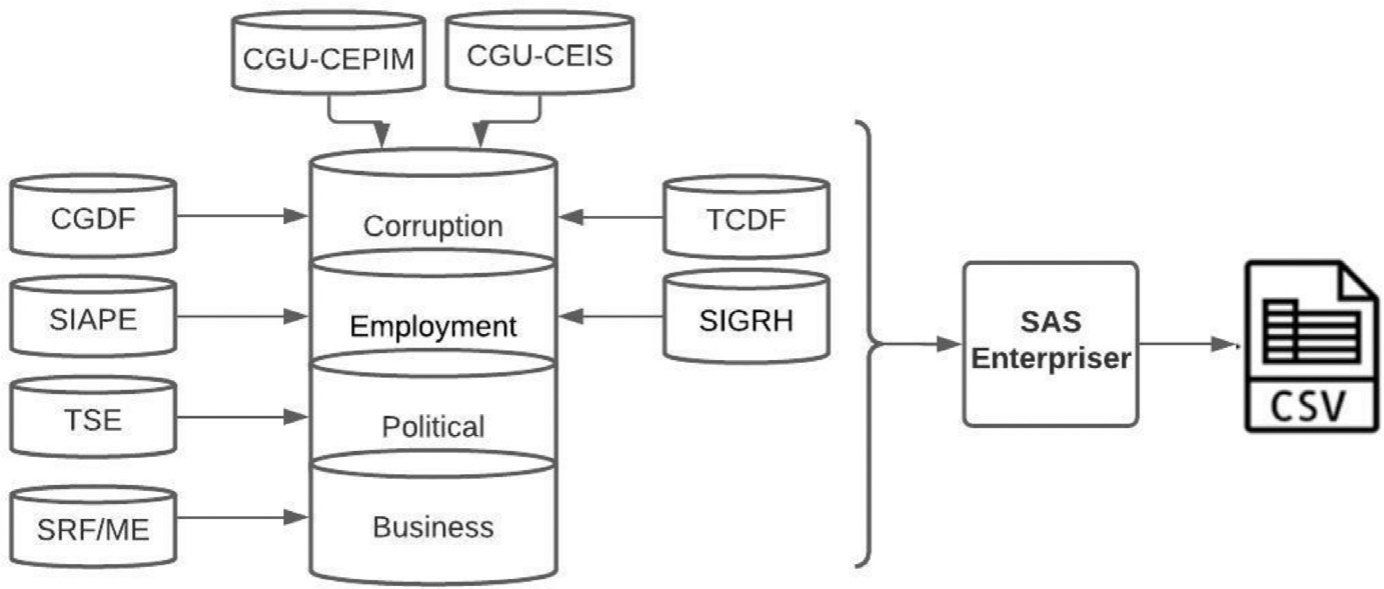
Illustrates the pipeline of ETL process (extract, transform and load) from different data sources integrated into a dataset aggregated by four domains and was submitted to a pre-processing (Data Enrichment and Data Cleansing).

These four domains (Fig. 1) are:

Corruption Domain (C) aggregates data corresponding to illegal acts committed by civil servants or militaries, or companies that they are owners.

Employment domain (E) is composed of base integration of two payment databases that have information from Public Security workers (policemen and firefighters) in Integrated Human Resource Management System (SIAPE) and from other civil servants (Education, Health, and other areas in Resource Management System (SIGRH).

Base integration work took place for the SIGRH, and SIAPE, since the same civil servant or military from the Federal District could be included in both databases due to the possibility provided by the Brazilian Federal Constitution to allow the accumulation of certain public offices.

Political Domain (P) has information from TSE, The Brazilian Superior Electoral Court, and provides information about candidates like level of education, party, marital status.

Business Domain (B) is composed of information from The Secretariat of Brazil's Federal Revenue - SRF/ME, about companies whose owners are civil servants and militaries.

### 2.3 Related literature

The decision about which attributes to compose this dataset was defined considering studies carried out on corruption literature, and all of them were identified and classified by previous domains defined (Table 2).

It is essential to bring the concept of corruption adopted for this dataset and represented by the variable "C.CorruptionTG". It was described in Brazilian Law No. 8429/92, which defines corruption as an act of improbity that, under the influence or not of the position, causes illicit enrichment, causes or not mandatory, will be used to the purse or violate Public Administration principles [20] and is described on Table 1.

**Table 1:** Attributes/features description.

#	Attribute name	Type	Brief Description
**Corruption Domain (C)**			

1	C.CorruptionTG	Boolean	Cases of dismission by corruption, this attribute could be a target for machine learning
2	C.CEIS	Boolean	Cases of individuals or legal entities with restrictions on the right to participate in tenders or to contract with the Public Administration by sanctions
3	C.TCDFrestriction	Boolean	Cases of person who are not qualified to exercise a position in a commission or a trust function within the Public Administration of the Federal District for a period of up to eight years due to serious irregularities found by the TCDF
4	C.CEPIM	Boolean	Cases of private non-profit entities that are prevented from entering into new agreements, on lending contracts or partnership terms with the Federal Public Administration, depending on irregularities not resolved in agreements, on lending contracts or partnership terms previously signed

**Employment Domain (E)**			

5	E.Salary	Numeric	Salary (Brazilian currency - Real) of the civil servant or military that included the salary received by any of the bases (SIGRH and SIAPE) or the sum of salaries in the case of civil servants who accumulate public positions as permitted by the Federal Constitution
6	E.SalaryMinusTax	Numeric	Salary with several discounts and obtained in a similar way to the "Salary" (SIGRH and SIAPE bases)
7	E.QtySIGRHOff	Int	Quantity of positions that the civil servant or military held until Nov/2020 into the SIGRH determined only with the SIGRH base.
8	E.QtySIAPEOff	Int	Quantity of positions the civil servant or military held in Public Security until Nov/2020 at SIAPE (Public Security, SIAPE)
9	E.QtySIGSIPOff	Int	Quantity of positions that the civil servant or military held until Nov/2020 in these two databases (SIGRH and SIAPE).
10	E.QtySIGRHfunc	Int	Quantity of functions that the civil servant occupied until Nov/2020 in the SIGRH (Servers, except Public Security, SIGRH)
11	E.QtySIAPEfunc	Int	Quantity of functions that the civil servant or military occupied until Nov/2020 in SIAPE (SIAPE Public Security)
12	E.QtySIGSIPfunc	Int	Quantity of functions that the civil servant or military occupied until Nov/2020 in these two databases (SIGRH and SIAPE)

**Political Domain (P)**			

13	P.ElectivePosition	Categorical	Type of electoral position that the civil servant disputed (president or vice, governor or vice, mayor, senator, councilor, federal deputy, state deputy, or district deputy)
14	P.CodParty	Categorical	Code of the party in which the server was registered for the election
15	P.CandElectiveSt	Categorical	candidate's registration status, which can assume the values 'Apt' (candidate able to go to the ballot box); 'Unfit' (candidate unfit to go to the ballot box); 'Registered' (registration of candidacy carried out, but not yet judged by the electoral body)
16	P.CandEducation	Categorical	Candidate's level of education can be defined as: non-disclosable, reads and writes, incomplete or complete elementary school, incomplete, or complete high school, and incomplete or complete higher education
17	P.CandMaritalSt	Categorical	The civil status situation of the candidate civil servant: single, married, non-disclosable, widowed, legally separated or divorced
18	P.CodRoundSt	Categorical	This attribute identifies the candidate's totalization situation in the turn that can be (elected, elected by average, elected by the electoral quotient, unelected, alternate, or null)
**Business Domain (B)**			

19	B.OwnershipPerc	Numeric	Percentage of share capital that the civil servant or military presents at Nov/2020
20	B.TypeOfOwner	Categorical	Type of partner of a civil servant or military is registered within the company to which it belongs
21	B.QtFirmAct	Int	Number of secondary activities registered by the company in which the civil servant or military is a partner
22	B.CodFirmAct	Categorical	The main activity of the firm/company in which the civil servant or military is a partner
23	B.CodFirmLegal	Categorical	Definition of legal nature of the company in which the civil servant or military is a partner, which may be in different denominations, such as: Mixed Economy Society, Public Limited or Closed Corporation, Limited Business Society, limited partnership, or by shares, among others
24	B.CodFirmSt	Categorical	Status of the company in which the civil servant or military is a partner, among the possible alternatives there are active, null, suspended, unsuitable, or closed
25	B.CodFirmSize	Categorical	Size of the company that can be Microenterprise (ME), Small Business (EPP), medium or large depending on the gross annual turnover of the head office and its branches, or that is, the global gross revenue defined in the tax legislation
26	B.DaysOwnership	Numeric	This attribute informs the number of days that the server is a partner in the company until Nov/2020
27	B.CodFirmTaxOpt	Categorical	This attribute informs if the company opted for the simplified taxation system - Simples Nacional - which aims to help micro and small companies concerning the payment of taxes

Source: An extract of this table was published on Vasconcelos et al. [4], p. 512/513 https://link.springer.com/chapter/10.1007/978–3–030–86230–5_40.

The Mendeley dataset is available at.

<https://data.mendeley.com/datasets/crpdknzswh/2>.

**Table 2:** Integer and numeric attributes.

Dim	Attribute	Min	25%	Median	75%	max	mean	std
E	Salary (Real BR)⁎	0.01	4651.28	7866.74	1464.63	40,140.21	9473.91	9752.79
E	SalaryMinusTax	0.00	3455.90	5388.11	8111.12	30,162.96	6725.79	8178.93
E	QtySIGRHOff	0.00	0.00	0.00	1.00	20.00	0.75	1.61
E	QtySIAPEOff	0.00	0.00	0.00	0.00	8.00	0.37	0.99
E	QtySIGRHSIAPEOff	0.00	0.00	0.00	2.00	20.00	1.12	1.85
E	QtySIGRHfunc	0.00	1.00	1.00	2.00	13.00	1.42	0.87
E	QtySIAPEfunc	0.00	0.00	0.00	0.00	4.00	0.03	0.23
E	QtySIGRHSIAPEfunc	0.00	1.00	1.00	2.00	13.00	1.45	0.91
B	OwnershipPerc	0.00	0.00	0.00	0.00	100.00	9.03	22.54
B	QtFirmActivities	0.00	0.00	0.00	1.00	11.00	0.79	1.81
B	DaysOwnership	0.00	0.00	0.00	499.00	43,795.00	917.40	2054.12

⁎ Brazilian currency - Real.

**Table 3:** Categorical attributes.

Dim	Attribute	Number of Categories	N° of examples
P	ElectivePosition	8	1317
P	CodParty	32	1317
P	CandElectiveStatus	5	1317
P	CandEducationLevel	6	1317
P	CandMaritalStatus	5	1317
P	CodRoundStatus	7	1317
B	TypeOfOwnership	2	86,058
B	CodFirmActivity	976	86,058
B	CodFirmLegal	33	86,058
B	CodFirmStatus	5	86,058
B	CodFirmSize	3	86,058
B	CodFirmTaxOption	5	86,058

**Table 4:** Boolean attributes.

Dim	Attribute	True	False
C	CorruptionTG	428	302,608
C	CEIS	132	302,904
C	TCDFrestriction	274	302,762
C	CEPIM	0	303,036

**Table 5:** Literature related to corruption.

DOMAINS	LITERATURE
Corruption (C)	Hanna and Wang [5], Carvalho and Carvalho [6], Carvalho [7]
Employment (E)	Gans-Morse et al. [8], Liou et al. [9], Carvalho [7] Padula and Albuquerque [10], Poocharoen and Brillantes [11], Carvalho and Carvalho [6] López-valcárcel et al. [12]
Political (P)	Pedersen and Johannsen [13], Bersch et al. [14], Meyer-Sahling and Mikkelsen [15], Moro [16], Carvalho et al. [17], Carvalho [7] Lassou and Hopper [18], Treisman [19], Gans-Morse et al. [8]
Business (B)	Carvalho [7]

The data obtained from these sources (Fig. 1) provided by different public organizations were aggregated in SAS Enterprise. They were outlined by their attributes classified by the four domains defined.

### 2.4 Pre-processing (Data Enrichment and Data Cleansing)

The data preparation is the stage in which the data must be processed and prepared in a way that can demonstrate the understanding of the business, in this case for corruption. Integrating different data sources could be a challenge because, in general, the data comes from sources of transactional systems or measurements or also from real-world situations, and the data set obtained must converge to understand the business.

Data cleaning and construction of attributes were carried out to generate treated and adequate data to enable the development of predictive models.

Data cleansing is the process of attempting to fill in missing values, smooth out noise while identifying outliers, and correct inconsistencies in the data [21]. It aims to alleviate two critical problems of data acquisition processes: the existence of missing values and the existence of noisy values (noise values).

The missing values occur when for the attributes of a dataset there is no determined value for some specimens or when a data set does not have values for an attribute of interest or even presents aggregated values concerning that attribute.

As a solution to the missing values, it was possible to remove observations with this characteristic, manually fill in values, or auto-fill.

The noisy values refer to changes from the original values and, therefore, consist of measurement errors or values considerably different from most of the other values in the data set, known as outliers. For example, we can mention cases that should be positive and negative values occur or a change in the behavior of the values of an attribute without explanation. Few observations were removed by specialist decision.

For the solution of noisy values, there is the inspection with the manual correction or automatic identification and cleaning implemented by algorithms that soften or cancel noise.

Data enrichment is the process of enhancing collected data with relevant context obtained from additional sources [22].

This dataset aggregates information from different databases that could benefit from a holistic approach. In addition, some features were elaborated in a specific way to provide information for business understanding.

The feature construction allows the elaboration of features that can generate relevant information according to the understanding of the business from the original data.

In this scenario, a feature construction was the transformation of categorical attributes into counting attributes.

This procedure was performed because the attribute, when expressing quantity, has meaning in the context of business understanding, while the categorical value does not express benefit in the context of corruption. For example, a categorical attribute that means the positions that the civil servant or military man/woman occupied in Public Administration has no meaning for this investigation. However, many positions he/she had occupied could inform that this one does not have a stable condition and could represent an anomaly.

It was applied for W.QtySIGRHOff, W.QtySIAPEOff, W.QtySIGSIPOff, W.QtySIGRHfunc, W.QtySIAPEfunc, and W.QtySIGSIPfunc from the Employment domain.

For machine learning research, it is essential to address the data imbalance problem. The relevant feature for research that should be the independent variable of this investigation (C.CorruptionTG) presents in the class of interest 428 records and in the dominant class 302,608 records, a relation that keeps the proportion of 1: 707, in percentage terms 0.14% of the class of interest in the population.

C.CorruptionTG is a dichotomous variable and is an important variable that has to be analyzed from other variables available for identifying risk factors that could be addressed to mitigate corruption in public administration.

Possible ways of dealing with this scenario are explained by Zhu et al. [23] that suggests solving the problem of learning on imbalanced datasets with two possible solutions: data-level solutions and algorithm-level solutions.

It is vital to inform that to comply with the GDPR legislation, the attributes that could identify a person were removed, making the data anonymized.

## Ethics Statement

The authors declare that they have observed all ethical requirements for publication in Data in Brief.

## CRediT authorship contribution statement

**Marcelo Oliveira Vasconcelos:** Conceptualization, Methodology, Software, Data curation, Writing – original draft, Visualization. **Luís Cavique:** Supervision, Writing – review & editing.

## Declaration of Competing Interest

The authors declare that they have no known competing financial interests or personal relationships which have or could be perceived to have influenced the work reported in this article.

## Footnotes

1 https://data.mendeley.com/datasets/crpdknzswh/2.
